# Linguistic signaling, emojis, and skin tone in trust games

**DOI:** 10.1371/journal.pone.0233277

**Published:** 2020-06-01

**Authors:** J. Jobu Babin

**Affiliations:** Department of Economics and Decision Sciences, Western Illinois University, Macomb, IL, United States of America; Rice University, UNITED STATES

## Abstract

This paper reports the results of an experiment involving text-messaging and emojis in laboratory trust games executed on mobile devices. Decomposing chat logs, I find that trust increases dramatically with the introduction of emojis to one-shot games, while reciprocation increases only modestly. Skin tones embedded in emojis impact sharing and resulting gains—to the benefit of some and detriment to others. Both light and dark skin players trust less on receipt of a dark skin tone emoji—suggestive of statistical discrimination. In this way, computer-mediated communication leads to reduced gains for dark-skinned persons. These results highlight the complex social judgment that motivates trust in an anonymous counterpart.

## Introduction

Computer-mediated communication (CMC) is rapidly becoming commonplace, a substitute to face to face, written, or voice communication. Communication is shown to help achieving cooperative outcomes, including face-to-face (FTF) communication (e.g., [1, 2, 3]), indirect (such as written) [4] [5]. More recent studies find that trust increases as agents communicate with complex, digital media, such as video game avatars [6], Facebook [7], and text chat [8], suggesting that digital modes may induce social utility comparable to FTF. However, as people increasingly employ CMC, studies have yet to focus on the role of emojis in an economic context.

Increasingly representative in digital discourse, emojis act as digital shorthand for expressions of emotions, ideas, and personae that enable users to sidestep the costs involved with saying something precisely with text [9, 10]. They are a common mechanism by which people chat, filter emails before opening them, and interpret consumer product reviews. Mobile devices now allow users to customize emojis by skin tone and gender, a commonly used feature pioneered by Apple in 2015. This is a significant development because economic interactions depend heavily on the perception of others.

Trust is an economic primitive—most exchanges remain unrealized without it. Ideally, such exchanges equate to enhanced social welfare. Yet, as people attribute behavior to a “type”, rather than to a situation, they may lower cognitive costs by stereotyping [11] or statistically discriminating [12]. People often make a decision about others based on some type of identifying signal that can be quickly read and interpreted. Identifying characteristics that have been shown to influence trust and reciprocity include: gender [13]; beauty and age [14, 15]; and skin shade [16, 17]. [18] use “friendliness icons” as stylized proxies for FTF interaction, observing that subjects in trust games prefer “friendlier” partners. That notion is supported across social psychology and computer science literatures (e.g., [19, 20, 21]). Some agents are willing to pay to supply, or acquire, identifying information in situations involving trust [22, 23], then opting to discriminate when given the chance to select a counterpart [24]. Such results underscore that choice of an emoji may serve as a valuable linguistic snapshot, impacting potential trust and reciprocity. The wrong signal may lead to unintended consequences.

In this paper, I explore how emojis impact trust-generated welfare and how skin tone markers—effectively signals—shape one’s willingness to trust. Exploiting a demographically diverse subject pool of mobile device users, I execute a laboratory experiment, exogenously restricting the type of computer-mediated chat players can use in a single endowment variant of the [25] “Investment game.” This game allows for the exchange of money in a manner that creates potential surplus (a growing pie), and captures intuitive measures of trust (money sent), reciprocity (a proportion returned), and social welfare (final payoffs). Student subjects may naturally select from a library of emojis that allow skin tone markers and can converse throughout the entirety of the trust interaction.

I find that trust increases dramatically with the introduction of emojis to one-shot interactions. Moreover, there is evidence that these effects are driven by conditional judgments of trustworthiness—essentially attempts to statistically discriminate. Trustworthiness is weakly affected; awarding trust yields poor returns on the margin. However, distribution of any gains is not even—dark skinned persons appear to be penalized for revealing their complexion. Persons receiving an emoji portraying a dark skin tone appear far less willing to trust to their counterpart. This result is common across agents of all skin tone types. However, all demographic groups ultimately act in a reasonably trustworthy manner that does not warrant such discrimination. I conclude that emojis impact the conditional judgment lying at the core of the trust relationship.

To my knowledge, this is the first paper to show how emojis serve as distinctive signals that help to drive, or dissuade, cooperative behavior. The sample is drawn from a pool of mobile-savvy college students at a demographically diverse public university. This is an advantage in that diversity unique to this sample allows an opportunity to observe signaling content in CMC and potential effect of associated stereotypes. Importantly, I limit the scope of my analysis to how the receipt of skin tone markers may impact trust and welfare. I do not address how specific emojis are chosen, rather, the effect of receiving one. I also do not explore the sequentiality of emojis in chat nor the effect of “complaints” about allocations. I focus on a single occurrence of a signal being received prior to a player choosing an action. The design is not meant to detect whether people misrepresent themselves strategically.

## Materials and methods

This paper explores how receipt of certain emojis impacts willingness to trust. The following experiment was carried out after the protocol was approved under “expedited” review by the University of Memphis Institutional Review Board, #PRO-FY2017-5. I obtained hand-signed informed consent forms. Subjects were privately paid, resigning the same forms as a receipt. The forms detail my protections to maintain anonymity and how a subject could exit at any time without penalty. No personally identifiable information was collected from any subject. Identifiers linking subject actions across the data were randomly assigned. Instructions, data, and full protocol for this paper are linked as Supporting information S1 File. See S2 Fig for a design flow diagram.

### The investment game

The Berg et al. [25] *Investment game* used in this paper (hereafter trust game) is a two player, one-shot sequential game of perfect information which has commonly been used to measure trust (extensive form given in S1 Fig). There are two roles: Investor (*i*) and Responder (*r*). The first-moving Investor gets endowment *e*_*i*_ = 100 and can send *M*_*i*_, which triples before reaching the Responder. The Responder in this study receives no endowment, *e*_*r*_ = 0.

A strategy *σ* for *i* is an amount to send, *M*_*i*_ ∈ [0, 100]. A strategy *σ* for *r* is to return an amount in [0, 300] with reaction function *k*_*r*_(3*M*_*i*_), after observing *M*_*i*_. With history *h*_1_, Player *i* chooses *M*_*i*_ to solve a payoff function

$$\begin{array}{c} \max_{M_{i}}\;U_{i}\left(M_{i},k_{r}\right)=\left[e_{i}-M_{i}\right]+k_{r}\left(3M_{i}\right)\;s.t.\;\;e_{i}=100. \end{array}$$

At *h*_2_, Player *r* solves

$$\begin{array}{c} \max_{k_{r}}\;U_{r}\left(M_{i},k_{r}\right)=3M_{i}-k_{r}\left(3M_{i}\right). \end{array}$$

Imposing sub-game perfection, the unique Nash equilibrium prediction is σ* = (*M*_*i*_, *k*_*r*_(3*M*_*i*_)) = (0,0). In reality, this equilibrium is seldom observed, with behavior indicative of players seeking Pareto improvements from the Nash. The realization of the strategy (100, 150) represents a socially efficient, “fair split” outcome (150, 150) that is also empirically rare.

The game yields intuitive measures of trust: amounts sent from the endowment = *M*_*i*_; trustworthiness: *θ*_*r*_% = *k*_*r*_(3*M*_*i*_)/*M*_*i*_, the proportion returned of an amount sent; and welfare = payoffs (an ordered pair in currency terms). However, a common complaint about the Investment game in this form is that it might capture some mix of trust and another prosocial behavior. Using the zero endowment version does have an advantage in that it makes analysis of what is returned and welfare gains more intuitive. The trade off is a potential distortion in the amounts sent when accounting for inequality aversion or “fairness” (see [26, 27, 28]). Also, Cox [29] argues that, while trust behavior in this game is “altruism plus,” a critical source of heterogeneity is individuals’ *expectation* of treatment by others. Expectations are important to this study.

### Experimental design

Three hundred ten undergraduate volunteers at the University of Memphis participated in a 1x3 between-subjects experiment over 7 sessions. Ad hoc power tests (*δ* > 0.5, power = 0.8, *α* = 0.05) indicated a target sample of 240-320 subjects. A session consisted of 30 to 87 subjects. Subjects arrived and signed a consent form. Each was seated spaced apart and instructed not to talk to others. Instructions were read aloud and projected onto an overhead screen prior to interaction, including how to install the instrument app onto a mobile device, open the experimental interface, and interact. All subjects used their own mobile devices after downloading the instrument app, guided by session assistants. As a priming device, subjects were given permission to text message or browse the Internet only until sessions began.

The instrument was the MobLab [30] mobile device interface, a cloud-based system for real time interaction. While this instrument is a departure from electronic lab standards, such as zTree [31], there are distinct advantages, such as a “taste of the field” and larger session sizes. Additionally, subjects are intimately familiar with device functionality.

In the first part of the experiment, subjects were randomly assigned to a CMC treatment, a role, and a counterpart to play a one-shot trust game under the following conditions:

- A baseline group (T1) plays a one-shot trust game with no communication allowed (control.
- A text treatment group (T2) plays a one-shot trust game with the ability to communicate via free flow, in-game text chat. No emojis are available. Subjects are not restricted by how many messages they may send.
- An emoji treatment group (T3) plays a one-shot trust game, in which the ability to communicate is restricted to emojis (without text). Subjects may naturally select from the full library available on mobile operating systems, as coded by Unicode developers and integrated into the MobLab app. Subjects are not restricted by how many they may send. The default emoji skin tone is yellow; subjects opt into modifiers to provide a different skin tone, based on the Fitzpatrick [32] scale. For more information, the reader is referred to Emojipedia.org. Emojis chosen may also contain gender modifiers and affective content.

Subjects were unaware of the different treatment conditions of the experiment. Previous studies have focused on “pre-play” messaging. In contrast, I allow a stream of communication throughout the interaction in T2 and T3. All players opt into chat in T2-3 and either mover can communicate prior to any decision by either. This allows for the second-mover to issue a response after observing an allocation. Upon the Responder’s action, the game ended and all CMC ceased. Study-wide, an interaction lasted a maximum of 5 minutes. The interface informed subjects of their in-play earnings.

In the second part of the experiment, subjects filled out a questionnaire post-interaction, conducted on the same mobile interface used for the game, collecting risk preferences, attitudes toward altruism/pro-social behavior, demographics, mobile device use information, and competency checks. Subjects were then excused, directed to a separate room to individually redeem the virtual earnings for cash at a ratio of 10 EC: US $1, plus a $5 participation fee. Once paid, subjects signed a payment receipt on their consent forms. The mean amount paid in cash for in-game earnings was $8.67 per subject, and the study paid out a total of $4425, including participation fees. There was no deception throughout.

The demographic inquiry excludes the traditional “ethnicity” questions. Instead, it included a question asking subjects to assess their skin tone using 4 tones, based on the Fitzpatrick [32] scale. This scale is used by Unicode developers to differentiate skin tone in emojis. Doing so ensured a light/dark delineation in subject responses, allowing me to match reported skin tone to any choice of emoji used in the T3. See S1 Fig, a concise breakdown of the subjects by gender and skin tone, detailing the balanced and diverse sample. A critical dimension of this inquiry, forty-four percent reported having a “brown” or “dark” skin tone. Forty-one percent were female (uniform across treatment groups). The vast majority (87%) of the subjects were aged 19-23. Cross platform matches presented the concern that images may not look exactly the same across different mobile platforms. Fortunately, 83% of the subjects in T3 used a mobile device with an Apple OS operating system. Only 2 dyads in T3 matched with different operating system. Five subjects raised concerns about technical limitations during a session. They were paid a $5 participation fee and dismissed.

Given the dearth of research, I have no priors for treatment effect size, especially with regard to trustworthiness. Thus, this study may be considered primarily exploratory. However, taking my cue from previous studies on trust and indirect communication (e.g., [5, 8, 18]), I expect that allowing CMC will result in increased trust. This would be reflected in the average amount sent (level of trust, ${\bar{M}}_{i,j}$) to be greater when communication is present, Furthermore, I expect to find some evidence that players to base actions on the identifiable characteristics of their counterparts (some degree of statistical discrimination), resulting in benefits to some types and penalties to others (e.g., [13, 16, 17]).

## Results

The first result is that there is a positive relationship between CMC and trust. Table 1 reports the means and standard deviations from the trust variables across treatments. Recalling the typically observed deviation from the SPNE prediction of zero amounts sent, average levels of trust increase steadily across the treatments. Mean amounts sent from the endowment in the baseline are 19.83 EC (s.e. 17.93), increasing to 35.54 EC (31.99) in T2, and increasing still to 47.13 EC (32.79) in T3. In both T2 and T3, the mean trust level is significantly greater relative to the baseline(*p* < 0.040 and (*p* < 0.001) respectively). Additionally, levels of trust between T2 and T3 are statistically different from one another (*p* < 0.049), suggesting that emojis have a different impact in the decision to trust than do pure text. See S4 and S5 Figs for bar charts of trust variables and payoffs by treatment and S1 Table for Mann-Whitney rank sum tests that demonstrate how distributions of trust variables differ.

**Table 1:** Mean, std. dev. of actions, payoffs.

	T1	T2	T3	All
Avg. Sent:	19.83 (17.93)	35.54 (31.99)	47.13 (32.79)	36.68 (31.09)
Avg. Prop. Returned (%):	74.90 (54.50)	81.20 (0.59)	105.00 (74.00)	91.70 (67.10)
Avg. Payoff:	71.76 (37.52)	90.51 (59.84)	94.65 (55.20)	86.68 (51.91)

*** = 1% los, ** = 5% los, * = 10% los

Means, s.d. for Sent and Payoffs are measured in experimental currency units, 10 EC: US $1. Means, s.d. of Proportion returned represent what is sent back from an amount sent. 301 subjects in study in 151 pairs. Sample is not balanced by treatment.

There is positive reciprocity on average in all treatments and increase, but the effect of CMC is less distinct. The baseline level of trustworthiness is 74.90% of what is sent (54.50), which is somewhat high relative to other studies. Text chat is associated with 81.20% of what is sent as a return (59.30). In T3, returns increases to 105% (74.00)—just above the threshold for “keeping the trust” where Responders give back as much as was initially sent. Trustworthiness increases only in T3 slightly relative to the baseline (*p* < 0.005).

There are welfare gains associated with CMC overall, but these gains do not vary between T2 and T3. However, the reasons for the gains in each treatment may differ if text and emoji function differently. Payoffs appear at their highest in the emoji sessions, 94.65 EC on average (55.20). It is even more evident that while levels of trust increase across treatments, the payoffs to first-movers do not. Mean payoffs increase sharply in both T2 and T3 relative to T1 for Responder, suggesting that trust does not result in a strong return for Investors. Overall, the bulk of any created surplus appears to go to Responders. I cannot rule out that some players may view their actions as being altruistic—being charitable by sharing their windfall—even though Responders would never have had positive surplus without the initial investment.

### Effects by skin tone

**Fig 1:**
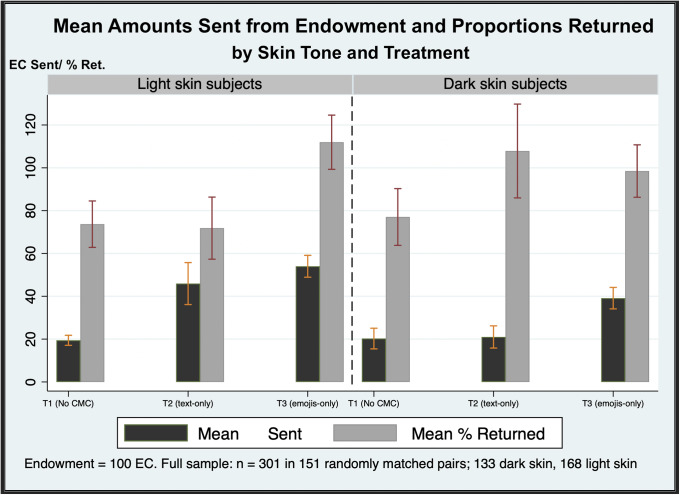
Mean trust variables, by treatment and skin tone. Data are the amount EC sent from endowment (trust) and the proportion returned from what is sent (trustworthiness). I report mean values with error bars.

Certain types of people have been shown to behave and respond differently in trust games. For example, Buchan, Croson, & Solnick [13] and Wilson & Eckel [15] report that females trust less readily on average, but tend to be more trustworthy (and thus, may be considered preferable counterparts). Similar findings for differences in skin tone are less clear. Fig 1 gives an illustrated breakdown of the effects of the two treatment conditions in this study and how they compare to the baseline of no communication.

I find evidence of group effects between the baseline and CMC treatments (all reported *p* values from rank sum tests). Light skin subjects trust more in the text treatment compared to the baseline (*p* = 0.002). However, I observe significant increases when emojis are used (*p* = 0.001). Similarly, dark skin subjects appear to trust more compared to the baseline (by about 15%, *p* = 0.002) with the introduction of emojis. Trustworthiness increases slightly for each skin tone group. Light skin Responders are about 30% more trustworthy in the emoji treatment compared to no chat baseline (*p* = 0.025). Dark skin players do appear to trust less than light skin in T3, a difference of about EC on average (*p* = 0.024). Dark skin Responders return about 20% more in T3, compared to the baseline (*p* = 0.003) and to some degree, act more trustworthy than light skin players in T2. Across the entire sample, dark skin players return 93% of what they are sent on average (s.e. 65.63) compared to 90% by light skin players (68.55). Thus, there is no kernel of truth to the belief that either group represents “bad partners.” Thus, any negative stereotypes seems misguided.

**Fig 2:**
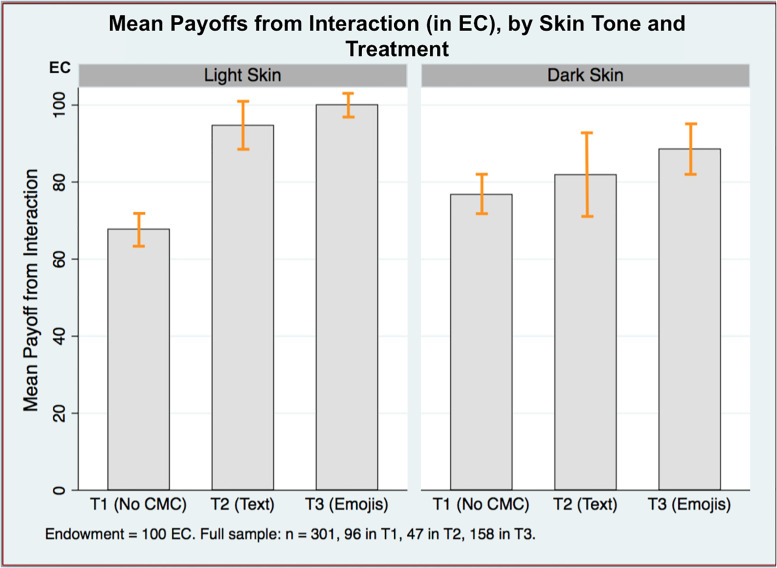
Mean payoffs, by treatment and skin tone. Data are the amount EC earned from interaction in trust game. I report mean values with error bars.

All players are better off with the introduction of CMC, yet Fig 2 reveals evidence that some groups of people benefit more than others. In the baseline where there is no CMC, dark skin subjects receive a higher payoff on average than light skin, 76.92 EC (47.29) compared to 67.90 (32.23), *p* = 0.004. This is likely the result of sending less from an endowment. However, light skin players see almost uniformly higher gains in T2 and T3 compared to dark skin when the ability to “reveal” oneself is available (rank sum tests confirm these differences). While payoffs increase for both skin tone groups in the emoji treatment (the locus of the most generated surplus), dark skin subjects received about 17 EC less on average compared to those with light skin (*p* = 0.011). These results suggest that, while both groups are better off overall in with communication, on average, dark skin players are worse off in the CMC treatments relative to the light.

In sum, I find sufficient evidence that the CMC treatments have a meaningful effect on trust and on trustworthiness, particularly in T3. These effects result in welfare differentials among players that communicate, consistent with the existing literature. In the following section, I explore how skin tone signals play a role.

### Analysis of T3: Emojis as signals

In this section, I am interested the effect of receiving a skin tine signal and how this impacts trust and welfare. To isolate these effects, I exploit the chat logs from T3, and I focus instances of players *receiving* an emoji *prior* to choosing an action. I code these emojis into indicator variables for skin tone, affective valence, and gender origin. I assume that once an emoji signal has been received, its informational content does not diminish. I also assume the choice of emoji involves time and cognitive costs.

Skin tone markers are a fairly recent development in emojis, allowing users modify an emoji with one of five skin tones. These tones are taken from the Fitzpatrick scale and formalized by Unicode 8 developers. Any emojis that do not use a tone modifier appear yellow, a default, neutral condition. Toned emojis are broken into two binary indicator variables: “dark emoji signal” (if an emoji fits Fitzpatrick Type- 3-5, medium or dark) and “light emoji signal” (if an emoji fits Fitzpatrick Type- 1-2, pale or light). I argue that these are signals, in that the player, at some point, would have had to actively switch from the default yellow.

To date, there is no formalized method to classify emoji by the *perception* of gender origin. To control for the perception of a female counterpart, I emulate the incentivized coordination games found in Houser & Xiao [33] and Babin [34] for natural content categorization by normative standard. One hundred ten student evaluators were tasked with gender classification of 120 emojis that were actually used in T3 and were paid $0.25 for correctly guessing the modal classification. Forty seven were given the designation “female emoji signal” or “male emoji signal.” The result of this task shows that certain emoji may be commonly *believed* to identify the gender of the sender (whether correct of not). To my knowledge, this method has not been used before to classify emojis in any capacity. Finally, I had the same evaluators determine whether a sequence of emoji reasonably constituted a “promise or deal” and include an indicator for such.

Scharlemann, Eckel, Kacelnik, & Wilson [35]; Eckel & Wilson [36]; Centorrino, Djemai, Hopfensitz, Milinski, & Seabright [37] find that smiles and frowns can impact trust. To control for affective content, I adopt results from [38]. Adopting their scale of emoji sentiment polarity, I classify each emoji used in this study as a “positive signal” or “negative signal” with a binary indicator. One downside of using the Novak et al. scale is positive bias; a disproportionate amount of emojis express positive sentiment.

There is no guarantee that any initiated conversation will get a response. Such is the case in only a few instances. The frequency of certain emoji types suggests that the Responders are readily providing signals (intentionally or not), which is sensible if they perceive that this information is important to the first mover. In T3, 521 total pictograms were received across 78 matched-pair chat logs, with an average of 3.32 emojis used per person (s.d. 2.40). Table 2 tabulates and breaks down the 269 instances of a particular emoji signal type being received by a player, *prior to committing to an action*. The critical emojis for analysis are 38 dark skin and 57 light skin. The relatively large number of “positive” emojis received is part due to coding of “polite” gestures that are commonly used to initiate conversation.

**Table 2:** Instances of emoji signals received by role in T3, pre-action.

Emoji Signal Received	by Investor	by Responder	Total
Dark Skin	17	21	38
Light Skin	38	19	57
Male	9	7	16
Female	19	21	40
Positive Sentiment	54	35	89
Negative Sentiment	17	12	29
Total	154	115	269

Emojis are selected by subjects from the full library on mobile operating systems as coded by Unicode developers and integrated into the MobLab app. Subjects are not restricted by how many they may send. All messaging ends at the second-mover’s action. Tabulations are restricted to all emojis a player received before committing to an action in the game.

### Effects of skin tone markers on trust

To determine the effects of emoji signals, I restrict analysis to the actual “treated” subjects found in dyads that opted to use emoji, with a trade off of diminished sample size. A post hoc power test confirms that the 158 subjects across 2 roles in T3 is above the lower threshold for significance with power = 0.8 and *α* = 0.05. Table 3 provides a breakdown of the basic characteristics of the subjects in the restricted T3 sample, which is balanced and diverse. Thirty-eight percent of these subjects are female and 48.10% reported having dark skin. Eighty-five percent percent reported using emoji in messaging every day while 86% of these subjects reported using a mobile device with an Apple OS. S1 File includes a summary of the T3 (restricted sample) statistics for the variables used in this analysis.

**Table 3:** Subject characteristics in T3, *n* = 158.

Characteristic	Female	Male	Total
Dark Skin (%)	32 (20.25%)	44 (27.85%)	76 (48.10%)
Light Skin (%)	28 (17.72%)	54 (34.18%)	82 (51.90%)
**Total** (*N*)	60 (38%)	98 (62%)	158 (100%)
Mean Age (s.d.)	20.40 (3.97)	20.34 (4.18)	20.34 (4.085)
Mean GPA (s.d.)	3.91 (0.77)	3.35 (0.48)	3.56 (2.35)
Uses text everyday	99%	97%	98%
Uses emoji everyday	89%	75%	85%

I assume that a signal does not not depreciate. That is, if an Investor receives an emoji indicative of a dark skin counterpart, this information is persistent. Following [39], I chose a Tobit model to minimize bias. Since payoffs are the result of dual action, I use OLS with robust standard errors to estimate marginal welfare effects. Table 4 reports Tobit estimations from the T3 emoji treatment and OLS estimates for welfare. I abandoned interaction terms due to collinearity and limited degrees of freedom.

**Table 4:** Marginal effects on trust decisions, T3, by emoji type.

	Variable Sent (EC) (Std. Err.)	Prop. Returned (%) (Std. Err.)	Payoff (EC) (Std. Err.)
Received light emoji	8.84	2.60	
	(10.52)	(27.80)	
Received dark emoji	-19.21***	-27.70	
	(8.98)	(33.20)	
Received male emoji	-9.11	33.70	
	(12.59)	(39.90)	
Received female emoji	20.21***	8.50	
	(7.89)	(12.00)	
Received positive emoji	5.26**	21.50*	
	(2.53)	(12.30)	
Received negative emoji	-23.60**	-38.70**	
	(9.92)	(16.90)	
Promises/deals made	11.86	15.19	17.48
	(10.45)	(14.10)	(12.42)
Emoji signal used in chat			16.43***
			(7.24)
Dark skin subject	-0.77	-36.20	-11.07***
	(18.13)	(21.10)	(4.14)
Female subject	-5.95	10.30	4.41**
	(13.53)	(20.10)	(2.22)
Risk tolerance score	-0.15	5.36	0.02
	(0.36)	(5.11)	(0.51)
Intercept	44.12***	3.98***	89.05
	(22.24)*	(1.74)	(26.28)
*N*	74	71	151
R^2^	0.187	0.198	0.194
LR *χ*^2^/*F*	21.12	23.24	18.20

*** = 1% los,

** = 5% los,

* = 10% los

Treatment effects of CMC on the treated T3 groups that actually used emoji. All received emoji variables represent signals received pre-action. *Risk tolerance* reflects a 1% increase in score on relevant scale. Tobit regression estimates reported for columns 1 and 2. Three observations top censored, 5 bottom censored. Estimates are measured in experimental currency units (EC), 10: US $1. Column 1 shows estimates for amounts sent, the trust measure. Column 2 give estimates for the trustworthiness measure, proportion returned. Column 3 shows OLS estimates for the welfare measure payoffs in EC using robust standard errors. The 71 Responders exclude those players that received zero from their counterparts.

The key result from this analysis is that trust is negatively associated with reception of a dark skin emoji, as the amount of the endowment sent reduces by more than 19 EC on average (s.e. 8.98). Estimating gains in T3, I find the average payoff going to dark skin players as 11 EC (s.e. 4.14) less than that of a light skin subject, mirroring what I observe in Fig 2. I observe no significant effect related to using a light skin emoji.

Restricting the sample to only include subjects reporting dark skin, I find that the negative effect of a dark emoji on trust is persistent (27 EC less sent on average), with the average payoff going to players is 9 EC (3.54). This suggests that dark skin emojis are salient and that the effect is common across both skin tone groups, a finding that contrasts with many studies on in group/out group cooperation. Spearman tests show dark and light emojis are negatively correlated (-0.349) while dark skin subjects and dark skin emojis are positively correlated (0.432) so I have no concerns about widespread misrepresentation. I conclude that it is likely that the observed payoff differences between skin tone groups are the result of discrimination.

When an Investor is faced with a female-oriented emoji, trust increases by 20 EC (s.e. 7.89, *p* = 0.002). The net gain in T3 for females on average is 4 EC (2.22). This observation supports the narrative in buchan2008trust: because females are perceived to be more trustworthy on average, they are preferred counterparts and thus, are more trusted than males. However, the emoji classification method is noisy and the reader is advised to view this result critically. Positive and negative sentiment emojis are predictably correlated with trust, consistent with the literature on facial expression detailed in aforementioned studies.

## Discussion

Eckel & Wilson [36] suggest that people do not view trust as a problem involving traditional notions of risk aversion (e.g., a financial gamble), but rather as one in which one’s choice of action is contingent on social judgment—trust is “socially risky.” Each agent in dyadic trust relationship—particularly the first-moving Investor—makes decisions based on expectations about the other. *a priori* beliefs typically guide these expectations, but so does any information obtained. When one decides to use an emoji they make a decision about how to position them self in relation to a counterpart. If people prefer certain types as counterparts, sending an emoji indicating a particular gender or skin shade or could be beneficial (or detrimental) to one’s hopes for a positive return.

The goal of this study was to identify how potential signals—skin tone markers in emojis—can affect trust behavior and impact welfare. There are three main takeaways from this inquiry. First, while communication fosters economic trust, emojis impacts trust differently from text, where the mechanism appears to be one of signaling and screening, rather than promises made and realized. I concede that since the Responder lacks an endowment in this design, I cannot distinguish between trust and concerns over fairness.

Second, because emojis have embedded signaling content, they have a strong effect on trust and resulting welfare gains. Knowing a counterpart’s “type” may enable the decision-maker to calibrate most likely responses. While some groups of people may be generally considered “preferable” counterparts in social games, players may choose emojis signaling their status as good partners. In this way, emoji-use has a significant welfare effect. Dark skin individuals appear to be paying a penalty as they reveal themselves with certain emojis: as trust decreases with a dark skin emoji, so do payoffs for those that send such signal. This appears to be the unfortunate result of a negative stereotype, although I do not elicit beliefs in this study.

Finally, if we observe attempts to statistically discriminate, beliefs are often wrong and don’t pay strong returns on the margin. In this study, dark skin players reciprocate at high levels, meaning that any underlying negative stereotypes are not founded in reality. Hence, the use of statistical discrimination is inefficient. I find no evidence that investors are adept at gauging the trustworthiness of Responders from their emoji exchanges. As a result, there can be distinct winners and losers when emoji signals are sent.

The sequential nature of the emoji streams in my chat logs are worthy of their own study. One potential concern in the regression results is that in order to establish a causal relationship, one must assume the emoji signals are correlated with the trust variable of interest and not one another. Because the binary dummies for the emoji signal types are taken from data that are collected over the duration of the agents’ interaction, there are collinearity and endogeneity issues. Such would be the case if players condition the emojis they send on the signals they have previously received.

The literature suggests that females are commonly considered preferable counterparts. In this study, women receive higher gains than men, over 4 EC more on average, possibly the result of using the female emoji. Investors send around 20 EC more when they see what I classified as a “female emoji signal.” A future study with a stronger foundation for revealing gender with emojis might strengthen this finding. Subjects may be apt strategically “switch” their identities, given what they know about a the distribution of groups in the subject pool (such as a dark skin subject strategically choosing to use a light skin emoji). I find little evidence that this occurs, yet, one can imagine existence of a pooling equilibrium in which everyone uses the trust-garnering emojis. Future studies may focus on such cases, as well as the agents’ willingness to pay for the right to send computer mediated messages or emojis.

## Conclusion

Written “text” form divorces electronic language from gestures, facial expressions, and characteristic features. Emojis are compact, digital, linguistic devices making information exchanges more efficient and richer. One’s choice of electronic “words” is inexorably linked to the “self” they choose project to another, and such projections influence economic actions. The results in this paper make it evident that emojis have intrinsic qualities that impact welfare differently than other forms of communication. A fundamental argument in this paper is that emojis can be noisy, but salient, signals from which agents form a conditional judgment. Thus, individuals using electronic messaging should be aware of unintended consequences.

## Supporting information

S1 Fig Extensive form of the trust game. I opt for a variant of the [25] Investment game in which the first-mover receives a 100 EC endowment, the second-mover receives no endowment. The unique sub-game perfect Nash equilibrium is {(0,0)}.
(TIFF)

S2 Fig Design flow and summary of treatments. Add descriptive text after the title of the item (optional).
(TIFF)

S3 Fig subjects by gender and skin tone group (full sample *n* = 301). A concise breakdown of the subjects by gender and skin tone, detailing the balanced and diverse sample.
(TIFF)

S4 Fig Mean trust variables broken down by role and treatment. Data are the amount EC sent (trust) and the proportion returned from what is sent (trustworthiness) in trust games. The bars represent mean values with error bars. Trust increases as each instance of CMC becomes available and amounts sent are at there highest with emoji-use. Trustworthiness is at its highest level in T3, yet on average, proportions returned are little more than what was sent initially.
(TIFF)

S5 Fig Data are the amount EC earned from interaction in trust game. The bars represent mean values with error bars. I observe average welfare steady overall for Investors, increasing only slightly in T3. Mean payoffs increase in T2 and T3, relative to the baseline.
(TIFF)

S1 File Details about experimental design, protocol, data set, and written instructions for experiment. Summary of statistics for analysis. Link: https://www.protocols.io/view/linguistic-signaling-emojis-and-skin-tone-in-trust-bf54jq8w. Data available at https://figshare.com/articles/trustdata/11900235.
(PDF)

S1 Table Results of Wilcoxian rank sum hypothesis tests. I compare equality of means for trust variable distributions across treatments. 301 subjects in study in 150 pairs, not balanced by treatment.
(PDF)
