# Supplementary figures and images for: Linguistic signaling, emojis, and skin tone in trust games
Source: PLoS One. 2020 Jun 1;15(6):e0233277. doi: 10.1371/journal.pone.0233277 (PMC7263582; doi:10.1371/journal.pone.0233277)

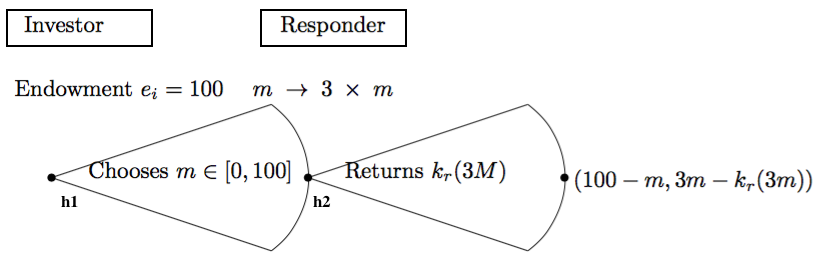

Supplement: S1 Fig — I opt for a variant of the [25] Investment game in which the first-mover receives a 100 EC endowment, the second-mover receives no endowment. The unique sub-game perfect Nash equilibrium is {(0,0)}. (TIFF) [file pone.0233277.s001.tiff]

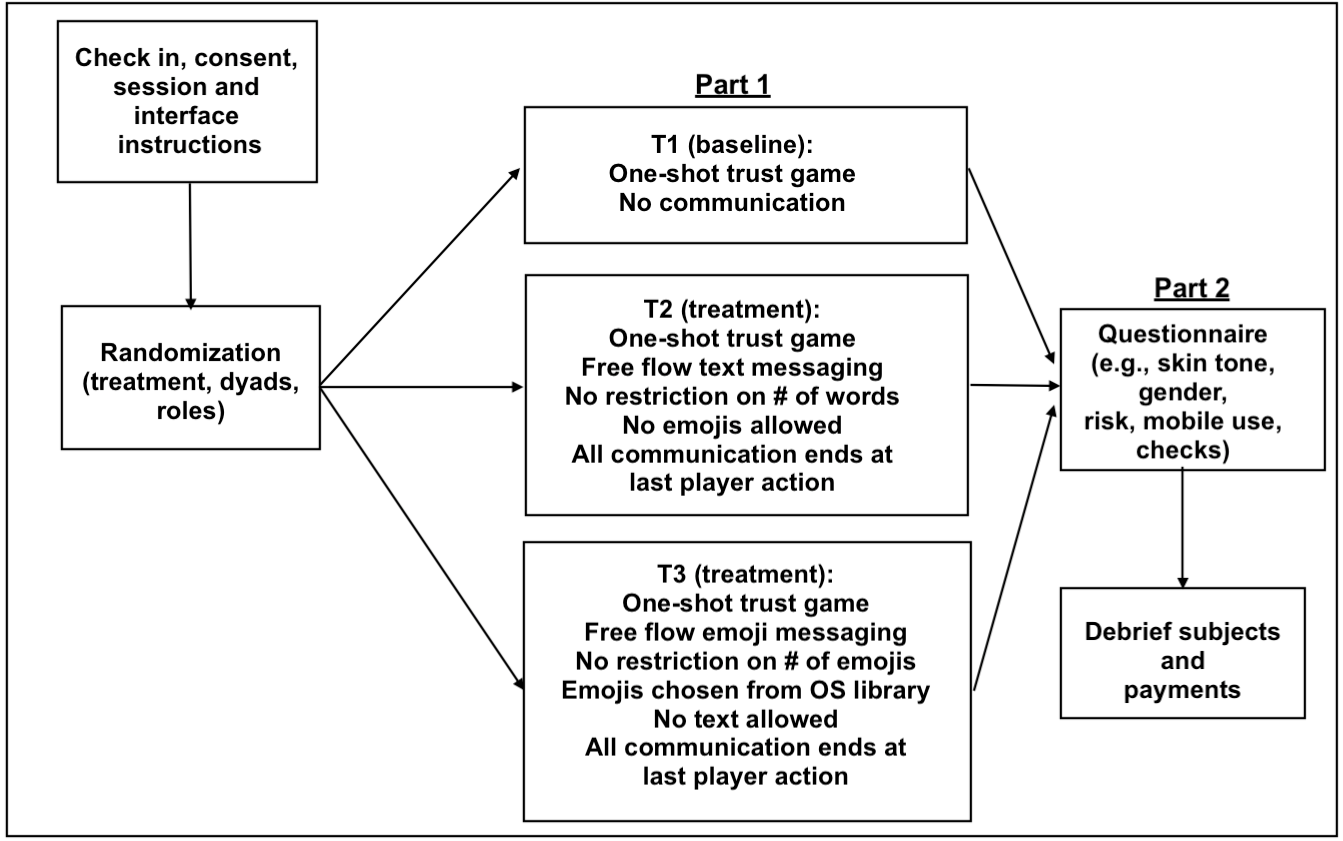

Supplement: S2 Fig — Add descriptive text after the title of the item (optional). (TIFF) [file pone.0233277.s002.tiff]

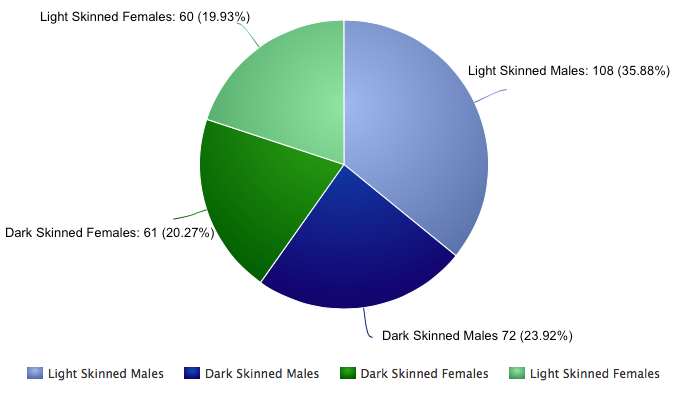

Supplement: S3 Fig — A concise breakdown of the subjects by gender and skin tone, detailing the balanced and diverse sample. (TIFF) [file pone.0233277.s003.tiff]

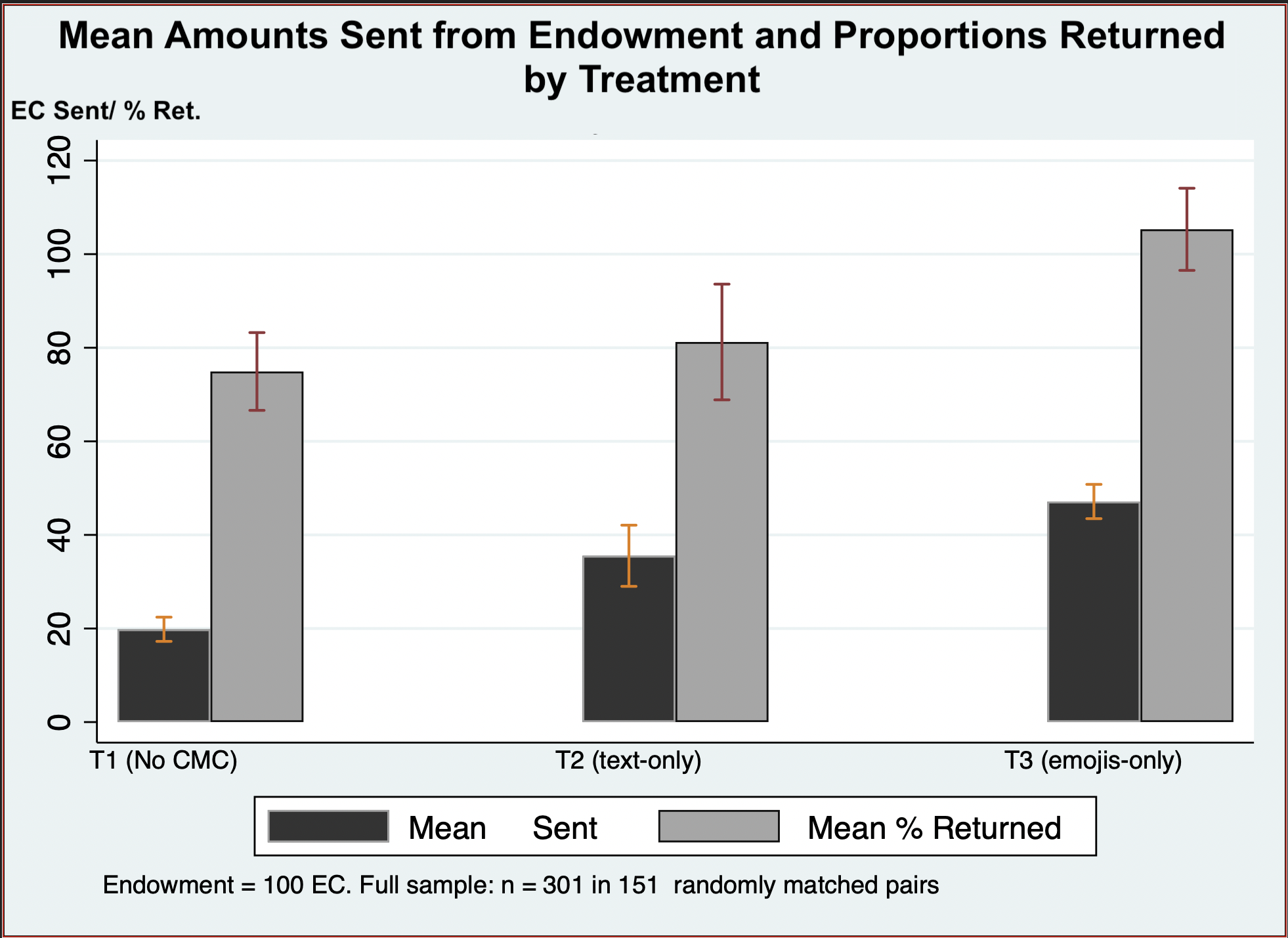

Supplement: S4 Fig — Data are the amount EC sent (trust) and the proportion returned from what is sent (trustworthiness) in trust games. The bars represent mean values with error bars. Trust increases as each instance of CMC becomes available and amounts sent are at there highest with emoji-use. Trustworthiness is at its highest level in T3, yet on average, proportions returned are little more than what was sent initially. (TIFF) [file pone.0233277.s004.tiff]

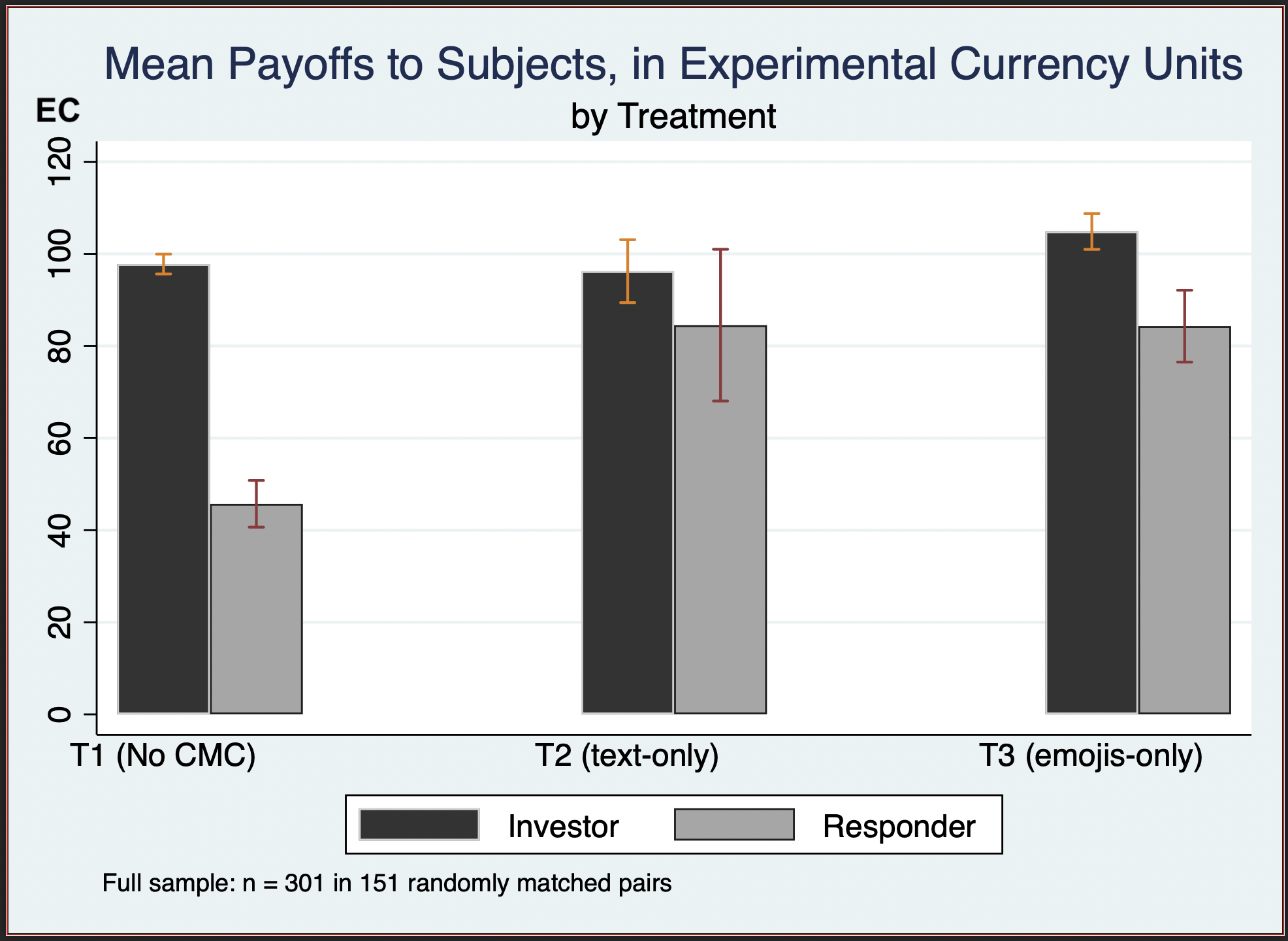

Supplement: S5 Fig — The bars represent mean values with error bars. I observe average welfare steady overall for Investors, increasing only slightly in T3. Mean payoffs increase in T2 and T3, relative to the baseline. (TIFF) [file pone.0233277.s005.tiff]
