# Supplementary material for: Linguistic signaling, emojis, and skin tone in trust games
Source: PLoS One. 2020 Jun 1;15(6):e0233277. doi: 10.1371/journal.pone.0233277 (PMC7263582; doi:10.1371/journal.pone.0233277)
Supplement: S1 File — Summary of statistics for analysis. Link: https://www.protocols.io/view/linguistic-signaling-emojis-and-skin-tone-in-trust-bf54jq8w. Data available at https://figshare.com/articles/trustdata/11900235. (PDF) [file pone.0233277.s006.pdf]

Linguistic signaling, emojis, and skin tone in trust games[†]

J.Jobu Babin[‡]

Abstract

This document provides the supplemental materials from an experiment involving text messaging and emojis in laboratory Investment games executed on mobile devices. I have included the session instructions for three treatments, images of the data collection instruments, sum stats tables, and a table listing debrief questions. This document is meant to supply the reviewer with a deeper sense of the methodology.

Supplemental Resources and Protocol

[†] Supplemental Materials

[‡] Department of Economics and Decision Sciences, Western Illinois University; 430N Stipes, 1 University Circle, Macomb, IL, 61455; Tel.: +1.309.298.1152; jj-babin@wiu.edu; orcid.org/0000-0001-6022-0089

Welcome

- Welcome to our study. We have some information for you before we begin.
- This study will be carried out on your mobile devices with the MobLab app. The class code for this study is **psrdri1u**.
- Let me ask you to refrain from talking your neighbors from this point on.
- However, you are free to use your device to complete any electronic messaging, before the session actually begins.
- Once the session has started, please **do not** use your device for anything else or exit the app.

Welcome

- We are inviting you to participate in an academic study of economic decision-making (IRB approval #2017-5).
- Our aim is to understand how people like you use information and technology to make certain decisions.
- It is completely voluntary, and you will be compensated for your completion. You may withdraw at any time.
- Please listen carefully to these instructions. Failure to follow instructions may result in your losing your earnings.
- If at any point in time you are unsure or have a question, quietly raise your hand and a session assistant will help you.

Consent Form

- In order to participate, you must complete a form acknowledging your consent.
- It describes the methods and procedures we have included in the design to protect you and ensure privacy. You cannot be personally identified from your responses.
- We utilize a double blind procedure, which ensures confidentiality of your responses from the researchers and participants. MobLab cannot record your IP nor track your mobile device ID.
- The study is not designed to impart a direct benefit to you personally. There is no more risk to you than you would face in everyday life as a student.
- Complete the form and return it to a session assistant. You will sign it again after the session, as a receipt for your compensation.
- Your compensation for completion is $5. However, you have the opportunity to earn "virtual currency" as part of the study, at a rate of V$10 - US$1.
- Your final payment can be redeemed for cash in 364FCB, on an individual basis, immediately after the session.

Reminders

- Thank you for completing your consent forms.
- Once I prompt you that the session is beginning, do not divert attention from the app. Do not use your device for any other purpose until the session is complete.
- Please listen carefully to these instructions. Failure to follow instructions may result in your losing your earnings.
- If you need assistance, please raise you hand and a session assistant will come to you.

Reminders

MobLab Class Code

- Please make sure that the MobLab app is properly installed on your device and that you have signed into your registered account.
- If you chose to use a laptop, navigate to MobLab.com and sign in to your account.
- It is important that you set the class code for the study on your device. Do this now by clicking **Join a class**.

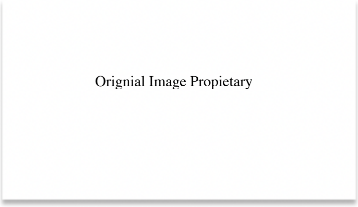

Enter **psrdri1u**

- Once you have joined, you should see a window for the study called **BA9000 Study**.

Instructions

Instructions 2

- The study consists of 2 activities: Part 1, an economic game and Part 2, a questionnaire that asks about you and the game.
- You will be prompted when to begin each component, and will see each appear in the app once started.
- Once each Part starts, you should briefly see a connection screen in the app:

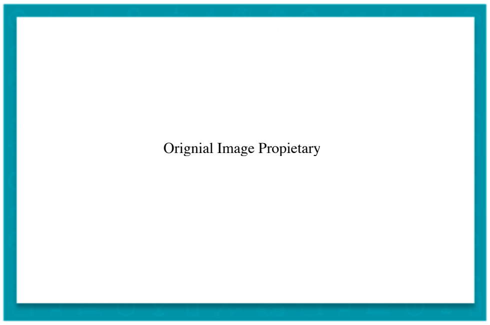

Instructions

- The session is now beginning.
- Please listen carefully to these instructions. Failure to follow instructions may result in your losing your earnings.
- Please do not use your device for anything else, nor should you exit the MobLab app without being asked to.

Instructions

- In this game, your earnings depend both upon the choices you and another make.
- There will be one round of play in the game.
- This round will last a maximum of 5 minutes, plenty of time to interact.
- There will be actions from which you choose, depending on your role.
- You will be randomly matched with another participant. No one will know with whom they are matched – your actions and responses are anonymous.
- Once matched, you will be assigned one of two roles, **Investor** or **Responder**, again randomly. The Investor will play first, followed by the Responder.

Instructions

- There will be a chat window available in your app. We encourage you to use it.

Orignial Image Propietary

- However, in this session, note that you DO NOT use emojis. We restrict your chat to text.

Instructions

- There will be a chat window available in your app. We encourage you to use it.

Orignial Image Propietary

- However, in this session, note that you DO NOT use text. We restrict your chat to emoji and numbers.

OpenMoji icons used in this figure (attributed below), recreated from the original image.
Subjects would see the proprietary version dependent on the operating system on their device.
During the instructions phase of a session, Apple versions of the emojis were viewed.

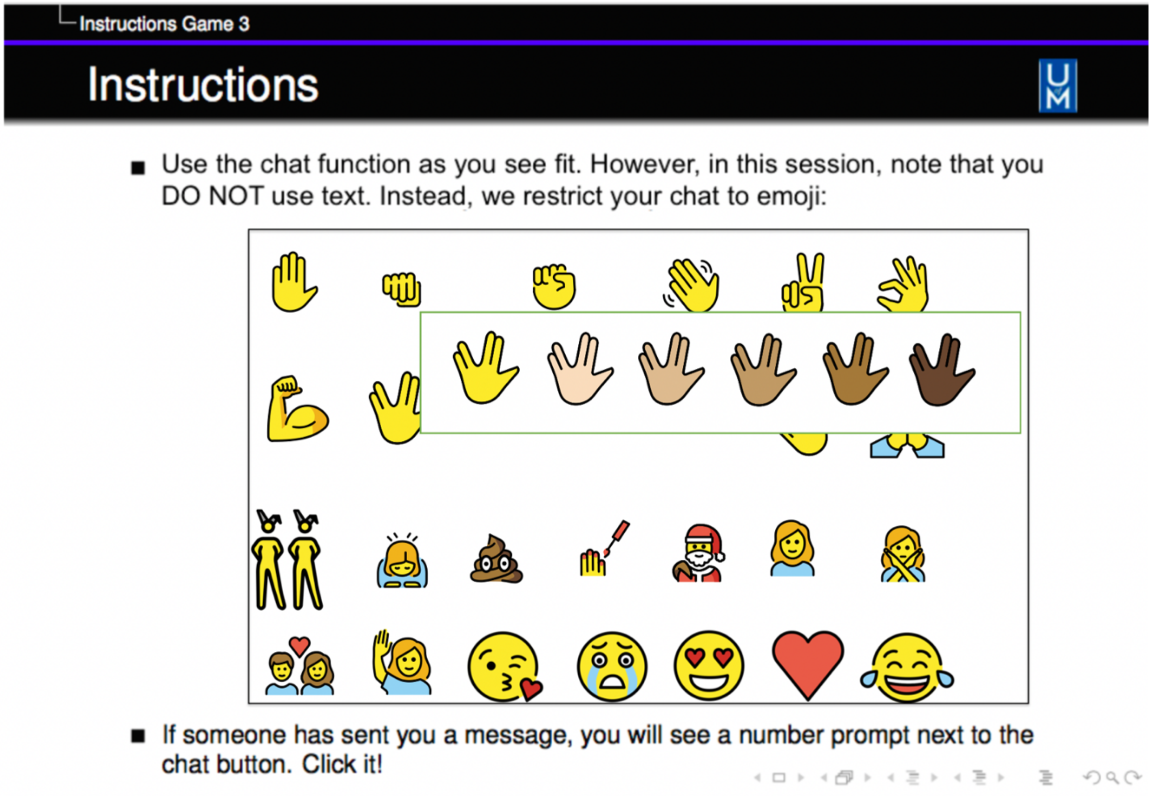

Fig: Instructions 10 – T3 Only (priming device)

Attribution: Emojis depicted are designed by OpenMoji – the open source and icon project.
License: CC BY-SA 4.0

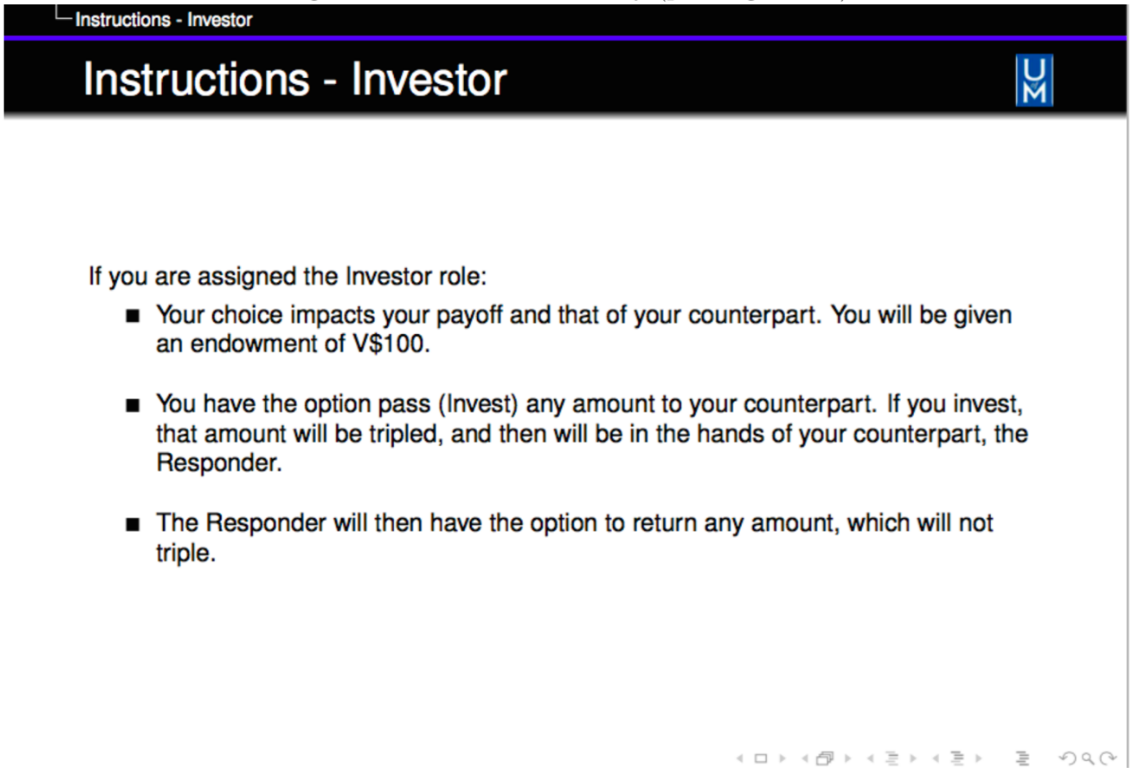

Instructions-Investor

Instructions - Investor

- You will see this interface when making your decision. Note the chat window, which is activated by the button at the bottom of the screen. You can use this function throughout the game.

Instructions-Investor

Instructions - Investor

- Move the slider to choose an amount of virtual currency to pass to the other player, which triples. To complete your action, click the Invest button. It will then be the Responder's turn.

At the end of the game, you will receive an earnings report, with the payoffs for each player. You will be asked questions about the game and yourself.

Instructions - Responder

If you are assigned the Responder role:

- Your actions impact your payoff and that of your counterpart. You **may** have been passed part of an endowment of V$100.
- If so, you have the option to return any amount you have to your counterpart.
- The amount that you return will NOT be tripled, and then will be back in the hands of the Investor.

Instructions - Responder

If you are assigned the role of Responder:

- Move the slider to choose an amount of virtual currency to return to your counterpart. To complete your action, click the Return button. The game will then be over.

Instructions - Responder

- You will see this interface when making your decision. Note the chat window, which is activated by the button at the bottom of the screen.

Instructions - Payoffs

- Once both players have completed their actions, the game is over.
- Each will receive and earnings report, summarizing the final earnings for each. You will be paid these in cash, at a rate of V$10 to US$1. In this example, an Investor would be paid US$11.60 plus the $5 completion payment after finishing the second component of the study.

- You may also see this amount by hitting the history button on the app.

Instructions-Part 2

Instructions - Questionnaire

- Once the games ends, you will be asked questions about the game and yourself.
- This will consist of another session on the app, and you will be prompted when to begin.
- There is a 15 minute timer for this part of the study. Please answer thoughtfully and honestly. If you finish early, please remain quietly while others finish.
- Once everyone in the session has finished the questionnaire, the study will be complete, and you are excused.
- To ensure confidentiality, your responses are encoded and no one will know them. Only your earnings will be reported.
- Your completion of both parts of the study will be verified by a researcher once you report to be paid.

Gender Origin Elicitation Instrument:

Attribution: emojis depicted are designed by OpenMoji – the open-source emoji and icon project.
License: CC BY-SA 4.0

		Gender Association		
		male	neutral	female
U+1F602	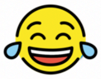	O	O	O
U+2764	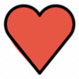	O	O	O
U+1F60D	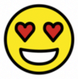	O	O	O
U+1F62D	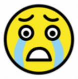	O	O	O
U+1F618	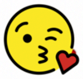	O11	O	O

Fig: IC Emoji Classification Instrument

This article focusses on "emojis" as being the set of Unicode characters that are displayed with color emoji presentation on operating systems.
This file contains names for The Unicode Standard, Version 7.0. Copyright © 1991-2014 Unicode, Inc. All rights reserved.

Table 2: Summary statistics (Full Sample)

Variable	Description	Mean	Std. Dev.	N
Treatment Variables				
Sent	amount $M_i \in [0, 100]$ sent by i ("trust")	36.68	31.098	151
Percent returned θ_r, j	percent of M_i returned by r ("trustworthiness")	0.9173	0.6713	137
Payoff	total payoff to player	86.68	51.920	301
Decision time	clock time of decision (0-300 seconds)	69.207	48.621	301
Text treatment (T2)	binary, 1=subject was in T2	0.156	0.893	301
Emoji treatment (T3)	binary, 1=subject was in T3	0.525	0.500	301
Cmc used	binary, 1=subject was in a dyad actually using CMC	0.299	0.458	301
Subject Characteristics				
Female subject	binary, 1=subject reported being female	0.403	0.491	301
Skin tone	Likert, Fitzpatrick scale	2.279	0.876	301
Dark skinned subject	binary, 1=subject reported dark skin tone	0.449	0.497	301
Age	in years	20.226	3.472	301
GPA	overall grade point average	3.49	1.738	301
Native English speaker	binary, 1=native English speaker	0..897	0.304	301
Subjects that chat each day	probability of text messaging any given day	0.977	0.149	301
Subjects using emoji each day	probability of emoji use any given day	0.819	0.409	301
Psychometrics				
Risk tolerance score	risk attitude index (100="I always take a risk")	53.742	21.062	301
Trust score	trust attitude score (100="people are always trustworthy")	46.06	14.50	301
Altruism score	altruism attitude score ("100=I am or should be altruistic")	48.366	13.742	301
Game Insights				
Actions as expected	degree counterpart acted as expected (100=completely)	55.701	33.379	301
Instructions clear	degree to which instructions were clear (100=perfectly)	0.981	0.247	301
Could detect partner identity	degree to which one could detect identity (100=completely)	55.701	33.379	301
T3 Emoji Signal Variables				
Received dark emoji	binary; agents receiving dark emoji	0.2789	0.383	158
Received light emoji	binary; agents receiving light emoji	0.3607	0.4817	158
Received male emoji	binary; agents receiving male emoji	0.1512	0.3027	158
Received female emoji	binary; agents receiving female emoji	0.2219	0.4166	158
Received positive emoji	binary; agents receiving positive emoji	0.6203	0.4868	158
Received negative emoji	binary; agents receiving negative emoji	0.1836	0.3884	158

Table 3: Summary statistics, T3 (Restricted Sample)

Variable	Mean	Std. Dev.	N
Investors	0.506	0.502	158
Responders	0.494	0.502	158
Amount sent	47.138	32.795	80
Percent returned	1.053	0.74	71
Payoff	94.656	55.208	157
Cmc used	0.937	0.244	158
Promise	0.0825	0.123	158
Subject Characteristics			
Female subjects	0.38	0.487	158
Skin tone	2.31	0.909	158
Dark skinned subjects	0.481	0.501	158
Age	20.399	4.085	158
GPA	3.563	2.358	158
Native English speakers	0.899	0.303	158
Subjects that chat everyday	1.025	0.821	158
Subjects that use emojis everyday	0.754	0.443	158
Subject with Apple devices	0.855	0.372	158
Psychometrics			
Risk Tolerance score	54.753	20.259	158
Trust score	47.934	15.663	158
Altruism score	49.466	15.423	158
Game Insights			
Counterpart actions were as expected	55.627	33.759	158
Instructions were clear	0.981	0.137	158
Degree to which partner identity could be detected	42.204	32.107	158
T3 Emoji Variables			
Emoji used in chat	0.963	0.233	158
Received dark emoji	0.177	0.383	158
Received light emoji	0.361	0.482	158
Received male emoji	0.101	0.303	158
Received female emoji	0.222	0.417	158
Received positive emoji	0.62	0.487	158
Received negative emoji	0.184	0.388	158

Table 4: Debriefing Survey Given to Trust Game Subjects

**Please give thoughtful and honest responses to the items in the questionnaire.**
**Your answers are confidential and will not be connected with you personally.**
**To what extent do each of the following statements accurately describe you?**
**Please indicate the degree to which you personally *agree* or *disagree***
**with each of the following statements by choosing from the scale below that reflects your opini**
**The sliders range from 0=strongly disagree to 100=strongly agree.**
In general, I am always ready to take a risk.
I am always ready to take a risk involving confidence in strangers.
I am prepared to take risks by sacrificing to improve my *future* benefit, rather than immediate concerns.
If I were to participate again, I would make more money
I tell lies to protect myself.
I tell lies to protect others.
People usually tell the truth, even when they know they would be better off lying.
Most students do not cheat when taking an exam.
It's only a rare person who would risk his own life and limb to help someone else.
If you want people to do a job right, you should explain things to them in great detail and supervise them cl
People pretend to care more about one another than they really do.
Most people would tell a lie if they could gain by it.
The typical person is sincerely concerned about the problems of others.
Most people are honest only because they are afraid of getting caught.
If you act in good faith with people, almost all of them will reciprocate with fairness toward you.
Most people exaggerate their troubles in order to get sympathy.
Most people would stop and help a person whose car is disabled.
People are usually out for their own good.
Most people lead clean, decent lives.
What percentage of the time did your counterpart act the way you had expected them to act in the game?
**The remaining questions have multiple response or open response choices.**
My age is: 1=under 22, 2=22 to 30, 3=over 30
My grade point average is approximately:
I use emoticons/emoji regularly in my everyday life.
I use text chat or electronic messaging regularly in my everyday life.
English is my first/native language. 0=no 1=yes
My overall skin tone is best described as: 1=very light, 2=light, 3= brown, 4=dark
My gender is best described as: 0=male 1=female
I am an organ donor. 0=no 1=yes 2=I don't know
I regularly give blood. 0=no 1=yes
The instructions for the session were clear. 0=no 1=yes
What type of device did you use to take part in the study? (open response)
Have you participated in this type of decision-making game before? 0=no 1=yes
What type of mobile device did you use to take part in the study (make, model)?
To what degree were you able to determine identifying characteristics of your counterpart.
For example, did you have reason to suspect your counterpart was a female, or of a particular ethnic group?
We are very interested in what you thought about the decision problem that you just completed.
In the space provided below, please tell us what kind of situation in your life this reminds you of,
or briefly explain your thought process. (open response)

Table 5: Debriefing Survey Given to Emoji Evaluators

**Please give thoughtful and honest responses to the items in the questionnaire.<br>Your answers are confidential and will not be connected with you personally.**
I understand the study's withdraw policy. 0=no 1=yes<br>My gender is best described as: 0=male 1=female<br>My age is: 18-100 slider<br>How many semesters of post-high school education have you completed.<br>In general terms, how would you describe your political orientation? 0-100, very conservative to very liberal<br>English is my first/native language. 0=no 1=yes<br>How frequently (during an average day, on a scale of 0-100) do you use a mobile device to chat,<br>instant message, email, or post tweets?<br>When using digital communication, how often do you use emoji in your everyday life<br>(during an average day, on a scale of 0-100)?<br>What operating system is on your primary mobile device?
Instructions (both treatments)
You are going to be asked to classify a series of 120 pictures into one of three categories,<br>according to a criterion you will hear in a minute.<br>There will be many others completing the same task you are.
IC Treatment
We ask that you pick the most appropriate answer. While there is not a correct answer to this question,<br>you can earn up to 2 $5 gift cards as a bonus for choosing the answer<br>you think will be the most common among everyone in the study.

Table 6: Summary Statistics for Subjects, Chapter 3

Variable	Mean	Std. Dev.	N
Duration	651.804	212.718	107
Treatment	0.505	0.501	107
Female	0.512	0.501	107
Age	23.304	7.216	107
Semesters	5.061	3.509	107
Native english	0.916	0.295	107
Texting Exp.	74.752	23.611	107
Emoji Exp.	56.434	31.197	107
Apple device	0.804	0.398	107

Table 7: Summary Statistics of Emoji Classifications

Variable	Mean	Std. Dev.	N
Observations	107.5	0.58	120
Mean	2.143	0.449	120
Std. Dev.	0.442	0.113	120
From male	0.167	0.374	120
From female	0.311	0.465	120
