# Supplementary material for: Linguistic signaling, emojis, and skin tone in trust games
Source: PLoS One. 2020 Jun 1;15(6):e0233277. doi: 10.1371/journal.pone.0233277 (PMC7263582; doi:10.1371/journal.pone.0233277)
Supplement: S1 Table — I compare equality of means for trust variable distributions across treatments. 301 subjects in study in 150 pairs, not balanced by treatment. (PDF) [file pone.0233277.s007.pdf]

S1 Table. Results of Wilcoxian rank sum hypothesis tests. I compare equality of means for trust variable distributions across treatments. 301 subjects in study in 150 pairs, not balanced by treatment.

Table 5. Mann-Whitney Tests for Treatment Effects.

	Sent (in EC)		Prop. Ret.		Payoffs (EC)	
Treatments	z-score	p-value	z-score	p-value	z-score	p-value
T1=T2	-2.018**	$p < 0.040$	-1.682	$p < 0.093$	-1.960*	$p < 0.049$
T1=T3	-5.031***	$p < 0.001$	-3.469**	$p < 0.005$	-2.754***	$p < 0.006$
T2=T3	-2.271**	$p < 0.049$	-0.816	$p < 0.414$	-0.581	$p < 0.562$

***=1% los, **=5% los, *=10% los
